# Herbivore grazing—or trampling? Trampling effects by a large ungulate in cold high‐latitude ecosystems

**DOI:** 10.1002/ece3.3130

**Published:** 2017-07-10

**Authors:** Jan Heggenes, Arvid Odland, Tomas Chevalier, Jörgen Ahlberg, Amanda Berg, Håkan Larsson, Dag K. Bjerketvedt

**Affiliations:** ^1^ Department of Environmental and Health Sciences University College of Southeast Norway Bø i Telemark Norway; ^2^ Scienvisic Linköping Sweden; ^3^ Computer Vision Laboratory Department of Electrical Engineering Linköping University Linköping Sweden; ^4^ FOI Swedish Defence Research Agency Linköping Sweden

**Keywords:** grazing, laser scanning, lichen, loss, reindeer, trampling

## Abstract

Mammalian herbivores have important top‐down effects on ecological processes and landscapes by generating vegetation changes through grazing and trampling. For free‐ranging herbivores on large landscapes, trampling is an important ecological factor. However, whereas grazing is widely studied, low‐intensity trampling is rarely studied and quantified. The cold‐adapted northern tundra reindeer (*Rangifer tarandus*) is a wide‐ranging keystone herbivore in large open alpine and Arctic ecosystems. Reindeer may largely subsist on different species of slow‐growing ground lichens, particularly in winter. Lichen grows in dry, snow‐poor habitats with frost. Their varying elasticity makes them suitable for studying trampling. In replicated factorial experiments, high‐resolution 3D laser scanning was used to quantify lichen volume loss from trampling by a reindeer hoof. Losses were substantial, that is, about 0.3 dm^3^ per imprint in dry thick lichen, but depended on type of lichen mat and humidity. Immediate trampling volume loss was about twice as high in dry, compared to humid thin (2–3 cm), lichen mats and about three times as high in dry vs. humid thick (6–8 cm) lichen mats, There was no significant difference in volume loss between 100% and 50% wetted lichen. Regained volume with time was insignificant for dry lichen, whereas 50% humid lichen regained substantial volumes, and 100% humid lichen regained almost all lost volume, and mostly within 10–20 min. Reindeer trampling may have from near none to devastating effects on exposed lichen forage. During a normal week of foraging, daily moving 5 km across dry 6‐ to 8‐cm‐thick continuous lichen mats, one adult reindeer may trample a lichen volume corresponding to about a year's supply of lichen. However, the lichen humidity appears to be an important factor for trampling loss, in addition to the extent of reindeer movement.

## 1 INTRODUCTION

Mammalian herbivores may be keystone species, generating vegetation changes with extensive top‐down effects on ecological patterns and processes (Cumming & Cumming, 2003; Holtmeier, 2015; Rosenthal, Schrautzer, & Eichberg, 2012; Suominen & Olofsson, 2000) and depending on herbivore size and habitat productivity (Bakker, Ritchie, Olff, Milchunas, & Knops, 2006; Cumming & Cumming, 2003). The ecological function, as well as behavior of in particular large herbivores in cold high‐latitude ecosystems, is commonly referred to as a single process, “grazing” (e.g., Albon, Brewer, O'Brien, Nolan, & Cope, 2007; Olff et al., 1999; Pellerin, Huot, & Cote, 2006). Grazing, however, involves at least two distinct and potentially different ecological effects, which usually are separate in theory, but rarely in studies. There is the eating of plants, and associated defecation and urination (e.g., Dove & Mayes, 1991; Holechek, Vavra, & Pieper, 1982; Stewart, 1967). Secondly, however, there is also the concomitant but less studied undirected trampling of plants, which may vary with season and be less, for example, when snow cover protects ground vegetation. Studies of trampling from warmer grasslands ecosystems, for example, from domestic species (e.g., Lezama & Paruelo, 2016; Ludvikova, Pavlu, Gaisler, Hejcman, & Pavlu, 2014; Olden & Halme, 2016) and from African savannas and grasslands (e.g., Cumming & Cumming, 2003; Dunne, Western, & Dietrich, 2011; Mudongo, Fynn, & Bonyongo, 2016), suggest trampling may have major ecological effects in herbivore communities, for example, on plant cover and composition, forage availability and foraging and food intake, and soil structure and associated nutrient cycling. A recent review concluded that trampling has an underestimated impact on plant species composition and richness (Rosenthal et al., 2012). The few studies that specifically address this process tend to focus on seed dispersal (Faust, Eichberg, Storm, & Schwabe, 2011; Horn, Pachmann, & Poschlod, 2013; Schulze, Buchwald, & Heinken, 2014; Wessels‐de Wit & Schwabe, 2010). For some high‐intensity use areas, by often domestic herbivores, the more obvious mechanical disruption of plant cover and soil, including soil compaction, infiltration of water, and nutrient cycling, has been studied (Drewry, Cameron, & Buchan, 2008; Ludvikova et al., 2014; Schrama et al., 2013; Xu et al., 2013). However, the more subtle effects on ground vegetation preceding the mechanical disruption of soil have rarely been studied (but see Van Uytvanck & Hoffmann, 2009; Cumming & Cumming, 2003; Plumptre, 1994). Although trampling is commonly referred to as important in studies of herbivore–vegetation interactions also in natural, cold, high‐latitude ecosystems, it is usually in an anecdotal and qualitative way (Austrheim et al., 2008; Suominen, Persson, Danell, Bergstrom, & Pastor, 2008; Persson, Danell, & Bergstrom, 2000; but see Pegau, 1970). There are methodological challenges on how to quantify such trampling. Here a novel method using high‐resolution 3D laser scanning enabled study and quantification of the resilience of plant cover to trampling, the associated functional basis, and environmental correlates.

Herbivore body size and hoof structure influence the number of imprints and area trampled (Cumming & Cumming, 2003). The most widely distributed mammalian herbivore is the cold‐adapted northern reindeer or caribou (*Rangifer tarandus* L.). This large ungulate with large hoofs and toes that can spread out survives in high‐latitude alpine and Arctic ecosystems (Skogland, 1983, 1984), which tend to be fragile and sensitive to disturbances (e.g., Körner, 2003). Reindeer live in herds, are almost constantly in motion, and graze extensively, and Arctic reindeer exhibit some of the longest ungulate migrations known, because of the typically low production and patchy distribution of high‐quality vegetation resources (e.g. Fancy, Pank, Whitten, & Regelin, 1989; Fryxell & Sinclair, 1988; Vors & Boyce, 2009). Therefore, reindeer grazing and presumably also trampling are major ecological factors that may reduce lichen abundance over large spatial areas (Holtmeier, 2015; Klein, 1968; Olofsson, 2006b; Suominen & Olofsson, 2000). Indeed, for lichen forage, a “wastage factor” of 2–10 times the food intake has been suggested (Gaare & Skogland, 1980; Vistnes & Nellemann, 2008), making it more important than grazing. Light lichen trampling, however, could have a positive effect on lichen population through increased clonal growth associated with lichen fragmentation and spreading (e.g., Kershaw, 1985).

Reindeer may forage extensively on lichens in both winter and summer (Hansen, Aanes, & Saether, 2010; Skogland, 1984; Vistnes & Nellemann, 2008). Slow‐growing ground lichens are favoured in snow‐poor (chionophobic) habitats with frost (Odland & Munkejord, 2008). Total lichen cover and thickness vary greatly. Dense lichen mats may reach a total dry biomass (DM) of 2 kg/m^2^ in undisturbed *Pinus* forests. In undisturbed alpine heaths, biomass is rarely more than 1.2 kg/m^2^ and 8–10 cm thick, and may be only from 0.2 kg/m^2^ (top) to 0.8 kg/m^2^ (bottom) on more exposed ridges. In strongly grazed (and trampled) sites, the biomass will often be lower than 0.1 kg/m^2^ (Odland, Sandvik, Bjerketvedt, & Myrvold, 2014). The most important reindeer grazing lichen belongs to the *Cladonia* genera. It includes numerous species, but only a few are abundant in current Western European wild reindeer areas, including *Cladonia alpestris*,* C. rangiferina* and *C. arbuscula*, as well as *Flavocetraria* spp. Lichens grow like a tiny, leafless, branching shrub (fruticose), like it has leaves (foliose), and are poikilohydric organisms (de Vries & Watling, 2008; Gauslaa & Coxson, 2011; Kershaw & Macfarlane, 1980). It indicates that the amount of water in the lichen podetia vary continuously with the air humidity. Humidity affects lichen pliability and elasticity. Increasing humidity may therefore confer increasing resilience to trampling damage, but this relationship is not well studied. Lichen are capable of surviving long periods in a desiccated state (Kappen & Valladares, 2007), but will be brittle, and most likely particularly susceptible to trampling when dry (Holtmeier, Broll, Muterthies, & Anschlag, 2003; Kumpula, Stark, & Holand, 2011).

Therefore, reindeer and lichen were chosen as a suitable interaction model in this experimental study, using high‐resolution 3D laser scanning to accurately quantify potential effects of hoof trampling. We hypothesized (1) that dessicated and brittle lichen would be very sensitive to trampling damage with no resilience to imprints and be an ideal worst‐case model for trampling. However, this would depend (2) on lichen species, (3) lichen mat thickness, and (4) lichen humidity.

## 2 MATERIALS AND METHODS

In a series of replicated (five replicates) factorial design laboratory experiments, with lichen mat type and humidity as categorical explanatory factors, each with three levels, and change in lichen volume as the continuous response variable, the effect of reindeer hoof imprints was tested and quantified by high‐resolution 3D laser scanning.

### 2.1 Lichen mat types

Lichen mats were all collected *in natura* by cutting 16 × 16 cm square samples from a natural, continuous lichen mats, using a specially designed square 20‐cm‐deep cutter made of 1.5‐mm tempered steel. A lichen sample was always down to the mineral soil. A separate flat steel piece undercut the sample. Three different types of lichen mats were sampled: (1) a thin (2–3 cm) mat consisting of the lichen species *Flavocetraria nivalis* and *F. cucullata*, representative of the lichen mats on top of raised ridges with no or little snow cover, (2) a thick (6–8 cm) mat consisting of the lichen species *Cladonia alpestris*, which usually forms pure mats, and (3) a thick (6–8 cm) mat consisting of the lichen species *Cladonia rangiferina* (and possibly *C. arbuscula* and *C. stygia*, often forming mixed mats, and the latter cannot be distinguished from *C. rangiferina* by looking at the mat from above), both representative of the richer lichen mats slightly lower on the sides of ridges and with more snow cover (e.g., Vistnes & Nellemann, 2008; Ferguson, Gauthier, & Messier, 2001; Holleman, Luick, & White, 1979). Sampling *in natura* was of pure lichen mats visually stratified by species and thickness. Maximum sample thickness and minimum sample thickness were measured and species composition estimated in percent.

### 2.2 Lichen humidity

Lichen samples were left to dry at room temperature for several weeks to be completely dry and brittle, which was the first level of the factor lichen humidity. The second level was 100% humidity, for which each sample was completely soaked in water for about 10 min (considered sufficient based on pilot experiments), and within a container to make sure no fragments were lost. The third level was 50% humidity, obtained by weighing each of the five replicates dry, then 100% wet, and averaging the weight difference across the replicates, and divided by two. The corresponding amount of water was sprinkled slowly over each sample, simulating rain using a colander with 1‐mm openings.

### 2.3 3D laser scanning

**Figure 1:**
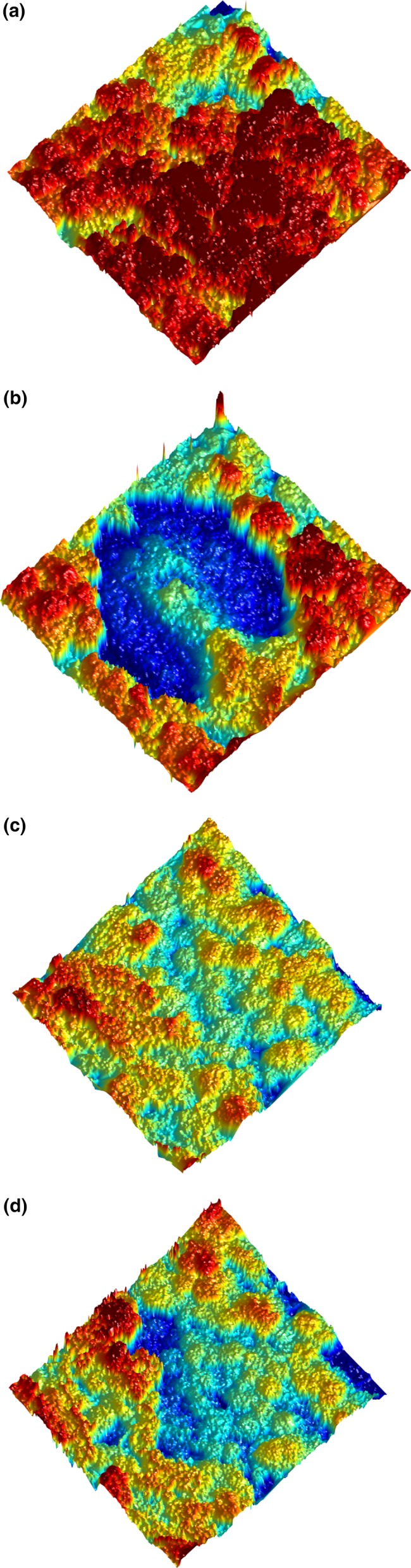
An example of a three‐dimensional view of a dry *Cladonia alpestris* sample and height estimation before and immediately after trampling. Top two: (a) untrampled and (b) trampled: dry lichen, bottom two: (c) untrampled and (d) trampled: 100 % humid lichen. Each square is 16 × 16 cm. The colors represent the height values

A fixed line scanner SICK IVP Ruler mounted on a 110‐cm high square aluminum frame was used for the laboratory measurements (SICK AG, Waldkirch, Germany; ftp://ftp.sickivp.se/download/Ruler%20E/Ruler_E_Reference_Manual.pdf; accessed 26 May 2015). The scanner illuminates the object below by a laser line (wavelength 660 nm (red), measurement swath width 600 mm, *x*‐*y*‐*z* axis resolution 0.6 resp. 0.45 and ~0.2 mm). The height profile was extracted by analyzing the line through a camera from an angled perspective. By electronically moving a table where scanning objects were placed, a sequence of profiles was extracted and assembled as a 3D model (Figure 1).

### 2.4 Experiments and analysis

Each trial consisted of five replicates across a combination of lichen mat type and humidity, that is, 3 mat types × 3 humidity‐level trials, with 45 samples in all. All five replicate samples were weighed and laser‐scanned before reindeer hoof imprints, to calculate dry weight and untrampled volume. Imprints were made using a natural reindeer hoof from an adult male (ca. 120 kg, hoof size ~10 × 12 cm) attached to a specially designed “hoof imprinter” consisting of a hoof holder, a control arm, and required weights. Hoof weight was standardized during experiments to 20 kg. A reindeer hoof normally exerts a pressure of 148–180 g/cm^2^ (Markgren, 1971), corresponding to about 20 kg for a hoof roughly 10 × 12 cm (160 g/cm^2^).

All samples were scanned immediately after one hoof imprinting (Figure 1), to calculate loss in lichen volume. Because elasticity may cause the lichen to regain volume as a function of time, laser scanning was repeated at fixed time intervals, with protocols depending on original humidity. The dry samples, not expected to regain significant volume, were rescanned after 1 hr as a control. Thereafter, they were soaked in water for about 10 min to gain 100% humidity, simulating heavy rains, and rescanned again to test whether humidity reduced lost volume. Wetted samples were also scanned immediately, and then rescanned after 1 hr, but with additional intermittent scans. The 100% and 50% humid samples were rescanned after 2, 5, 15, 30, and 60 min, respectively, amounting to about 100 scans in all. For logistical reasons, it was not always possible to follow the time intervals to the exact minute. It took 3–4 min to trample the five boxes in each set of samples, which contributed some variation.

The raw data were in a binary number format convertible to gridded height maps (Figure 1). The resolution of each pixel was 0.6 × 0.475 mm, with one height value in each pixel. Due to the perspective difference between the camera and laser line setup, one or more of the edges in each box was to a small extent shadowed from measurement. Loss of measurement points due to the geometrically scattered structure of the lichen is unavoidable. These apparent “losses” were treated with an interpolation process, primarily using the cubic spline method, supplemented with robust nearest neighbor interpolation when needed. To compare the volumes in each sample over time and estimate volume differences, the height data were (semiautomatically) registered, using translation and rotation, for maximum overlap. A maximum valid ROI (region of interest), individual for each sample, was then estimated.

R version 2.14.2 (Venables & Smith, 2012) was used for statistical analysis with ANOVA models for the experiments and repeated‐measures models for comparisons of changes in responses over time. The assumption of homoscedasticity was checked by Levene's test.

## 3 RESULTS

Data were lost for one pretrampling measurement of one replicate for *Cladonia alpestris* with 50% humidity. This replicate was not analyzed further.

### 3.1 Volume lost to trampling

Type of lichen mat and humidity both strongly affected volume immediately lost to trampling (multiway ANOVA; mat type: *p* < .0001, *F* = 20.8135, *df* = 2, 35; humidity: *p* < .0001, *F* = 48.9360, *df* = 2, 35; Levene, *p* = .3913, *F* = 1.0926, *df* = 8, 35). There was also a weaker interaction effect (mat type × humidity: *p* = .0018, *F* = 5.3728, *df* = 4, 35). Dry lichen lost considerably more volume than humid lichen (Table 1), and in particular for the thicker mats. Trampling loss was about twice as high in dry compared to humid thin (2–3 cm) lichen mats, and about three times higher in dry vs. humid thick (6–8 cm) lichen mats (Table 1). There was, however, no significant difference in volume loss between 100% and 50% wetted lichen (ANOVA with Tukey, humidity: *p* < .0001, *t* > 5.417 resp. *p* = .983, *t* = 0.180) or between the two different‐by‐species types of thick lichen (Table 1; ANOVA with Tukey, lichen type: *p* < .0158, *t* > 2.926 resp. *p* = .993, *t* = 0.035).

**Table 1:** Immediately lost lichen volume (dm^3^) by reindeer hoof trampling, and regained after 1 hr, depending on type of lichen and humidity. Means with *SE* in parentheses across five replicates

	Lichen thin (2–3 cm) *Flavocetraria* spp.		Lichen thick (6–8 cm) *C. alpestris*		Lichen thick (6–8 cm) *C. rangiferina*	
	Lost (dm^3^)	Regained (dm^3^)	Lost (dm^3^)	Regained (dm^3^)	Lost (dm^3^)	Regained (dm^3^)
Dry	−0.116 (0.030)	−0.002 (0.001)	−0.335 (0.031)	−0.0298 (0.031)	−0.322 (0.021)	−0.011 (0.004)
50% humidity	−0.063 (0.009)	0.007 (0.002)	−0.092 (0.025)	0.008 (0.006)	−0.114 (0.011)	0.047 (0.008)
100% humidity	−0.052 (0.004)	0.045 (0.002)	−0.119 (0.037)	0.115 (0.002)	−0.119 (0.026)	0.101 (0.012)

**Figure 2:**
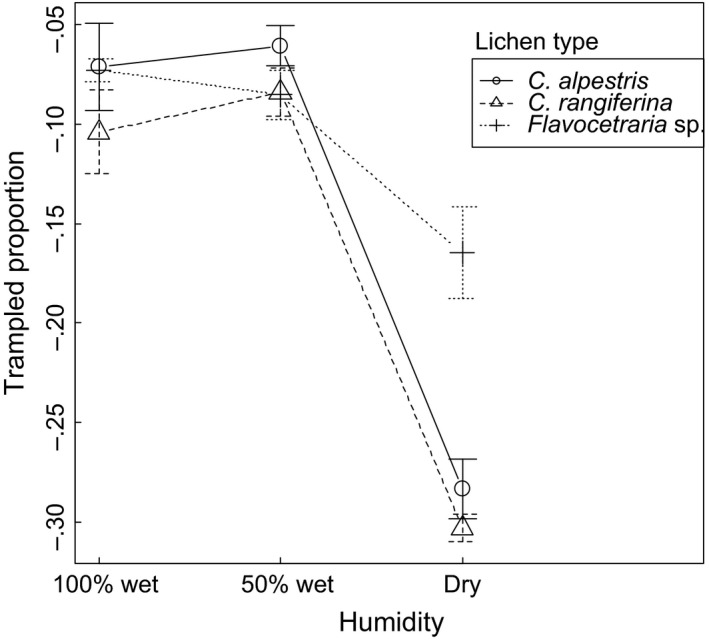
Means across five replicates per lichen humidity level (symbols) and *SE* (bars) of proportion of immediate loss in lichen volume caused by reindeer hoof trampling in different types of lichen mats

Because of the different thickness among the three lichen type levels, relative immediate losses in total sample volumes (proportion expressed as percent) were also compared. The relative loss of dry lichen was greatest for the two thick lichen levels (6–8 cm of *Cladonia alpestris* resp. *C. rangiferina*) with around 30% loss of volume within the lichen squares (Figure 2). There was no detectable difference between the two thick‐mat lichen species. The dry, thin (2–3 cm) type of lichen (*Flavocetraria nivalis* and *F. cucullata*) lost more than 15% volume after trampling (Figure 2). For humid lichen, however, interlichen differences vanished, and relative immediate loss of volume was reduced to 5%–10% (Figure 2).

### 3.2 Volume regained with time

**Figure 3:**
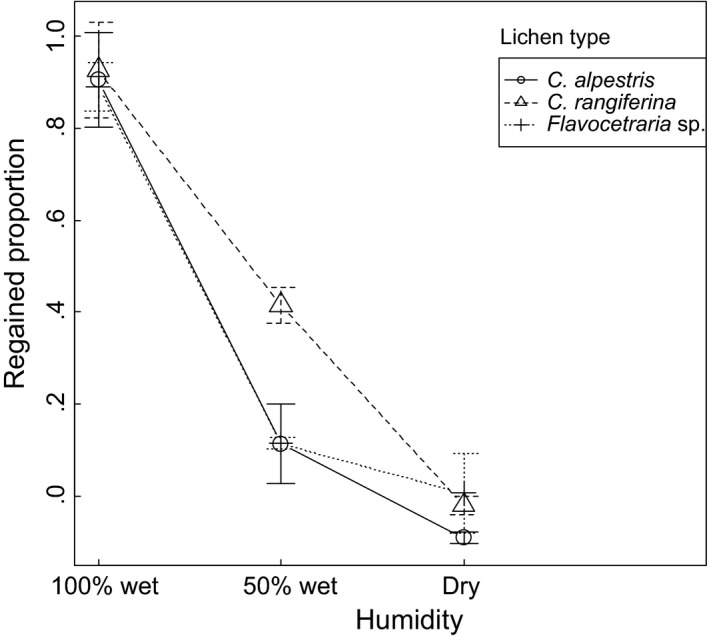
Means across five replicates per lichen humidity level (symbols) and *SE* (bars) of proportion of immediate lichen volume loss regained after 1 hr, for lichen volume loss caused by reindeer hoof trampling in different types of lichen mats

**Figure 4:**
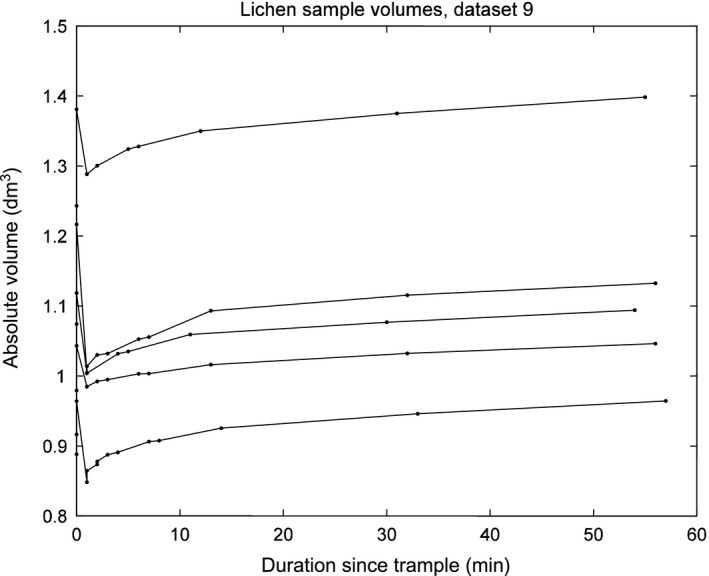
An example of volume data over time. Each line represents one sample of the five samples of thick *Cladonia rangiferina* lichen mat that were 50% wetted

Delayed measurement protocols varied somewhat depending on lichen humidity, but all samples were scanned after 1 hr and are directly comparable. Trampled volumes were reduced after 1 hr (dependent *t* test; *p* = .0002, *t* = 4.001, *df* = 43; Levene, *p* = .0758, *F* = 1.9986, *df* = 8, 35 for regained volumes), but depending on lichen mat type and humidity. Regained volume was insignificant for dry lichen regardless of lichen type (Figure 3, Table 1), 50% humid lichen regained substantial, but somewhat variable volumes, and less than the 100% humid lichen which regained almost all lost volume (Figure 3, Table 1). Therefore, humid *Cladonia* sp. lichen exhibited high elasticity, which conferred high resilience to reindeer hoof trampling. Most of the volume was regained after only 10–20 min, and regained volume by time decreased progressively thereafter (Figure 4).

The dry, trampled samples that were soaked in water for about 10 min to gain full humidity, simulating heavy rains, were also rescanned to test whether this reduced lost volume. However, dislodged and shattered fragments and general lichen swelling created laser beam “shadows” and confounded the volume results. If lichen height was used as a proxy for trampling effects (excluding *Cladonia alpestris* samples), dry trampled lichen regained height (*p* < .0001, pairwise *t* = 7.01, *df* = 10), but the effect size was small (from mean 36.67 to 38.17 mm). The height was measured on a 1 × 1 cm area (22 × 18 pixels) in the center part of the back end of the left clove (Figure 1).

## 4 DISCUSSION

Reindeer hoof trampling caused major volume losses of lichen, with important ecological implications, depending on the thickness of the lichen mat and especially lichen humidity. There were no detectable differences between the two thick lichen mat *Cladonia* species, but there was an interaction lichen mat type × humidity. The thick, dry *Cladonia* spp. mats lost relatively more volume to trampling than did dry, thin *Flavocentraria* mats, but for humid lichen the loss was smaller and similar. The ecological implications of trampling damage on exposed vegetation include a reduction in vegetation cover, biomass, and diversity (e.g., Holtmeier, 2015; Koster, Berninger, Koster, & Pumpanen, 2015; Olofsson, 2006b; Suominen & Olofsson, 2000). Lichen and moss cover are important in the structure and functions of these ecosystems via insulating and filtering effects controlling energy fluxes (Gornall, Jonsdottir, Woodin, & van der Wal, 2007; Peltoniemi et al., 2010; Stoy, Street, Johnson, Prieto‐Blanco, & Ewing, 2012). A well‐developed lichen mat strongly affects the moisture and thermal soil regimes. Its high reflectivity and low thermal conductivity reduce the heat into the soil, dampening diurnal temperature fluctuations and lowering soil temperature during summer (Kershaw, 1985). The lichen mat maintains soil moisture at or near field capacity and reduces drought stress (Bonan & Shugart, 1989; Kershaw, 1985). Trampling reduces lichen mat thickness, fragments and dislodges lichen, and shatters them into small fragments (Pegau, 1970), less suitable for grazing and much more susceptible to wind and water erosion (Belnap & Gillette, 1998; Pegau, 1970; Wielgolasky & Kjelvik, 1973). Lichen fragmentation, in turn, can play a role in lichen dispersion and clonal growth (e.g., Kershaw, 1985). Trampling and grazing may ultimately drive tundra ecosystem state transitions (van der Wal, 2006).

### 4.1 How much lichen volume is trampled?

Trampling effects depend on stride frequency, foraging time per day, and number of herbivores (hoof area; Hobbs & Searle, 2005; Cumming & Cumming, 2003). In dry lichen, volume losses were permanent and substantial, particularly in the 6‐ to 8‐cm‐thick mats, that is, more than 0.3 dm^3^ per hoof imprint. This would correspond to a trampled volume of about 1 dm^3^ lichen forage per step by an adult reindeer moving across a thick, dry lichen mat, allowing for some step overlap depending on pace and movement speed. One m^2^ of ca. 7‐cm‐thick *Cladonia* spp. lichen mat *in natura* weighs about 800 g. DM (Odland et al., 2014), meaning that 1 dm^3^ DM *Cladonia* spp. lichen weighs slightly more than about 10 g. An adult reindeer may have different stride lengths depending on pace and speed, but for walking, the stride length may be about 100 cm. Reindeer typically have bouts of activity and inactivity alternated across the 24‐hr day throughout the year (van Oort, Tyler, Gerkema, Folkow, & Stokkan, 2007). Daily accumulated movement distances will vary considerably, but may be, for example, 4–6 km in winter and 10–17 km in summer (Reimers, Tsegaye, Colman, & Eftestol, 2014). If moving 5–10 km with stride length 1 m across exposed continuous dry 6‐ to 8‐cm‐thick lichen mats and trampling about 1 dm^3^ per step, as indicated in the present study, this would correspond to trampling and compression of a lichen volume of 5,000–10,000 dm^3^, or 50–100 kg, by one individual in 1 day. The by far largest wild reindeer population in Western Europe, on Hardangervidda, Norway, has been managed based on lichen as a limiting resource (Gaare & Skogland, 1975; Mysterud & Austrheim, 2008). These reindeer each likely consume around 450 kg DM of lichen per year (2.1 kg DM per day, winter season 220 days; Bjerketvedt, Heggenes, & Odland, 2015; Holleman et al., 1979). Therefore, our results indicate as a worst‐case scenario that during one dry summer week, a reindeer moving a likely distance of 5 km or more across continuous 6‐ to 8‐cm‐thick lichen mats could trample a lichen volume corresponding to a year's supply, or more, of lichen forage. By the same reasoning, a flock of 100 reindeer would only need to move 500 m across a dry lichen mat to trample a year's supply for one individual. This is, however, an overestimate, as step overlap likely would be considerable. Also, caribou may be selective feeders (e.g., Mathiesen et al., 2000) and the amount of damage may be less in terms of amount, although probably not any less in terms of food lost. Furthermore, thick lichen mats may be rather uniform and continuous in some parts of alpine and Arctic ecosystems, but such landscapes often consist of a mosaic of different vegetation patches. Thus, for assessment of trampling loss in an actual landscape, the results presented here should be combined with a vegetation map. Obviously, if covered by snow in winter, trampling effects will vanish, depending on thickness and quality of the snow cover. However, the slow‐growing ground lichens are favoured in snow‐poor habitats with frost, typically on elevated wind‐blown ridges (Odland & Munkejord, 2008; Sundstoel & Odland, 2017) which may be exposed even in winter. The dry lichen's sensitivity to trampling damage makes them an excellent model for a worst‐case trampling scenario, and lichens are important forage for the most widespread large ungulate, reindeer, in the vast and fragile alpine and Arctic ecosystems. Damage on vascular plants will likely be less, and also depends on a number of additional environmental factors, for example, plant cover, height, species, brittleness.

### 4.2 The importance of quantifying trampling

Although invariably considered important (e.g., Boudreau & Payette, 2004; den Herder, Kytoviita, & Niemela, 2003; Kumpula, Kurkilahti, Helle, & Colpaert, 2014), to the best of our knowledge no other recent studies attempt to quantify the trampling effects on lichen. Nevertheless, for example, a recent reindeer winter grazing study by Vistnes and Nellemann (2008) refers to an “estimated spillage factor of 10” in Gaare and Skogland (1975). Presumably spillage includes trampling and food spill. If this was the case, the ecological effects of how reindeer behave and move, that is, causing spill and trampling, would be far more important than how they graze. The focus on grazing in relevant literature (e.g., Kumpula et al., 2014; Moen & Danell, 2003; Olofsson, 2006a; Olofsson, Moen, & Ostlund, 2010) may appear somewhat distorted. The food requirements and diet of reindeer are extensively studied and research methods well established (e.g., Gaare & Skogland, 1975; Holleman et al., 1979; Ophof, Oldeboer, & Kumpula, 2013; Storeheier, Mathiesen, Tyler, Schjelderup, & Olsen, 2003), as are the extent and consequences of active grazing by reindeer (e.g., Gaio‐Oliveira, Moen, Danell, & Palmqvist, 2006; Kumpula et al., 2011; Tommervik, Bjerke, Gaare, Johansen, & Thannheiser, 2012). However, considering the repeated suggestion that trampling is so important, that there actually may be a spillage and trampling factor of up to 10 (Gaare & Skogland, 1975; Vistnes & Nellemann, 2008), this ecological process is conspicuously understudied. We are only aware of one rather anecdotal quantitative report. In a pilot study based on measurements of trampling and material removed, but not eaten by six domestic reindeer on relatively thick lichen mats (not specified), Gaare and Skogland (1975) reported a loss factor of 10, relative to what the animals actually consumed during the snow season (103 g ±*SD* 71 dry matter (DM) m^−2^ versus 10 g DM m^−2^). It was later modified to a “wastage” factor of 2–10 (Gaare & Skogland, 1980), attributed to reindeer selective feeding, but not trampling. In an early observational field study in an open Arctic tundra system, Pegau (1970) herded approximately 500 reindeer over an unused portion of a large dwarf shrub meadow during a rainy, foggy day and a dry, warm day. It was concluded that “on summer ranges where lichens comprise at least 30% of available forage, at least 15% of the lichens should be considered as unavailable because of trampling by reindeer.” Also later, some wastage estimates have been used in population/bioeconomic models. They demonstrate the importance of lichen, but with little concrete observational or experimental support for wastage factors, and mainly for forested, that is, less exposed ecosystems presumably more resilient to trampling effects. Moxnes, Danell, Gaare, and Kumpula (2001), with reference to Gaare and Skogland (1980) (above), used a nonlinear wastage factor function with a “relative loss” value ranging from 0.5 to 4.5 depending on density of lichen. In winter, the wastage factor may be much reduced especially in lower, forested areas with considerable snow depth, for example, to a factor of 1.3 in bioeconomic models (Tahvonen, Kumpula, & Pekkarinen, 2014; with reference to Moxnes et al., 2001). (Pekkarinen, Kumpula, & Tahvonen, 2015; with reference to Moxnes et al., 2001) included different wastage factors for forest ecosystems in winter = 1.3, spring = 1.6, summer = 3.0, and autumn = 1.6.

### 4.3 Trampling effects depend on moisture

The results presented here give quantitative support to the contention that trampling may be a major cause of lichen volume losses (e.g., Koster et al., 2013; Kumpula et al., 2011; Olofsson, 2009; Pegau, 1970). However, our results demonstrate that loss depends crucially on lichen humidity. The potentially severe negative trampling effects appear to be limited to trampling during dry weather periods. In 50% and 100% humid lichen, the negative effects of trampling nearly vanished. The water content in poikilohydric lichen increases rapidly on contact with liquid water, whereas rates of water loss are slower (de Vries & Watling, 2008). Optimum humidity for growth is between 40% and 70%, but lichen tolerates irregular and extended periods of severe desiccation, for up to 9 months for some species (Kershaw, 1972, 1985; Nash, 2008). In this dry state, lichens can survive wide extremes of temperature, radiation, and drought in the harsh environments they often inhabit, but will be vulnerable to reindeer trampling. Because lichens have no special water storage organ, they have little control over the status of their hydration. A dry lichen can quickly absorb from 3 to 35 times its weight in water (Kershaw, 1972, 1985; Nash, 2008), for example, during rainfall. The loss of water vapor to the air may occur rapidly during warm and dry days (Brown, 1963). However, it is uncertain how much water is required to obtain sufficient pliability to resist trampling in the terricolous lichen studied here.

In conclusion, trampling leading to lichen volume loss can be substantial during dry weather periods, with as much as 0.3 dm^3^ per hoof imprint and consequently about 1 dm^3^ per reindeer step. However, lichen humidity is a key factor, as trampling volume loss nearly vanishes in wet weather. In a climate perspective, the predicted warmer climate in wild reindeer areas (Hanssen‐Bauer et al., 2015) does not bode well for the distribution and production of lichen. However, climate predictions are also for wetter weather, which likely will reduce potential lichen losses due to trampling. Regardless, the local movements and area use by wild reindeer and dry weather–dry lichen periods are the important factors controlling lichen forage volume loss by trampling.

## CONFLICT OF INTEREST

None declared.
